# In silico thermal control of spiral wave dynamics in excitable cardiac tissue

**DOI:** 10.1016/j.bpr.2024.100170

**Published:** 2024-07-02

**Authors:** Rupamanjari Majumder

**Affiliations:** 1 Nantes Université, INSERM, l’institut Du Thorax, Nantes, France

## Abstract

Self-organizing spiral waves of excitation occur in many complex excitable systems. In the heart, for example, they are associated with the occurrence of fatal cardiac arrhythmias such as tachycardia and fibrillation, which can lead to sudden cardiac death. The control of these waves is therefore necessary for the treatment of the disease. In this letter, I present an innovative approach to control cardiac arrhythmias using low (nonfreezing) temperatures. This approach differs from all previous established techniques in that it involves no drugs, no genetic modification, no injection of foreign bodies, no application of voltage shocks (high or low, single or pulsed), and no curative damage to the heart. It relies on regional cooling of cardiac tissue to create a transient inhomogeneity in the electrophysiological properties. This inhomogeneity can then be manipulated to control the dynamics of the reentrant waves. This approach is, to my knowledge, the most sustainable theoretical proposal for the treatment of cardiac arrhythmias in the clinic.

## Why it matters

Fatal cardiac arrhythmias are often associated with the occurrence of self-organizing, sustained spiral and scroll excitation waves. If left untreated, these waves disrupt the electromechanical pumping function of the heart and impair cardiac output, leading to sudden cardiac death. Controlling these waves is therefore essential for the treatment of these life-threatening diseases. Here, I present an innovative approach to control cardiac spiral waves without the use of drugs, genetic modifications, biocompatible (foreign) particles, voltage shocks, or curative damage to excitable cardiac tissue. This approach uses local temperature modulation to create transient inhomogeneities that can be used to control the dynamics of reentrant waves. It is by far the most sustainable approach proposed theoretically for treatment of arrhythmias in the clinic.

## Introduction

Self-organizing spiral excitation waves occur in many complex excitable chemical (1,2), physical (3), and biological (4) systems. These waves exemplify the emergence of order in systems that spontaneously transfer disorder to their environment (5). Spiral waves occur 1) as single rotating waves characterized by one or more fundamental frequencies (6), 2) as bound or unbound pairs with similar or opposite chirality (7), 3) in large numbers whose dynamics are dictated by a single driver (the mother rotor) (8), or 4) as wavelets exhibiting chaotic spatiotemporal patterns (9). In each case, the patterns occur as potentially self-sustained. Thus, understanding the dynamics of these waves is therefore of utmost importance for establishing control over the complex excitable dynamical system.

In the mammalian heart, the occurrence of spiral waves is of particular clinical importance, as these are associated with fatal cardiac arrhythmias, namely ventricular tachycardia, ventricular fibrillation, and atrial fibrillation (AF) (10,11). Since the mid-twentieth century, the conventional (and very effective) method used for treating ventricular tachycardia and ventricular fibrillation has been defibrillation (12). It involves administering a strong electric shock to the heart (O(10J) for internal devices and O(100J) for external devices) (13). Conventionally, defibrillation uses the high-energy shock to terminate all ongoing cardiac electrical activity and ensure total electrical resynchronization. However, the treatment is extremely painful, traumatic, often damaging toward the heart tissue itself, and associated with other negative side effects such as mental disorders and depression (14). As a result, there is a huge demand for alternative treatment: in particular, innovative medicine, low-energy therapeutic control, and further-optimized conventional cardiac defibrillation. Some of the significant advances made in this direction include low-energy antifibrillation pacing (15), various approaches using optogenetics (16), ultrasound ablation (17), sonogenetics (18), engineered heart muscles (19), biological self-restoration (20), hydrogel electrode injection (21), etc. Each approach has its specific set of advantages and disadvantages, which limits its applicability to particular cases, depending on the type of substrate and the type of arrhythmia under consideration.

In this letter, I demonstrate, for the first time, real-time thermal control of spiral waves in cardiac tissue. My approach is based on the use of temperature to induce a local, transient change in the electrophysiological properties of cardiac cells in specific regions of the heart. This study is motivated by my previous research using optogenetics (22), where I demonstrated real-time manipulation of spiral waves by optical “dragging” of its organizing center or core. Here, I replace optogenetics by using low temperatures (regional cooling) to achieve similar control over the spiral and scroll waves, eliminating the need for genetic modification of the heart and overcoming the problem of poor light penetration in deep tissue, which are the two main drawbacks of optogenetics.

It has long been recognized that ambient temperature has a profound influence on cellular and molecular processes (23,24,25,26,27,28), as it directly affects the rate constants of gating and the reaction rates of certain subcellular chemical processes. These, in turn, influence the kinetics of ion channels. Consequently, a change in ambient temperature leads to altered morphology and characteristics of the action potential as well as intercellular communication. In cardiac electrophysiology, the effect of temperature is included in the kinetic equations by dividing the activation and inactivation time constants of an ion channel by a temperature-dependent scaling factor Q(T). Equivalently, the rate constants of the respective subcellular chemical reaction can be multiplied by Q(T). According to the Arrhenius law of heat transfer, Q(T) has a power-law dependence on temperature (25,29). Previous studies show that decreasing temperature leads to an increase in resting membrane potential (RMP), a decrease in action potential amplitude, and a prolongation of action potential duration (APD) in rabbit and ground squirrel cardiomyocytes (30). It has also been reported that electrical signal conduction velocity decreases with decreasing temperature (31). These effects are similar to the effect of light on cardiac tissue in optogenetics. Therefore, I hypothesize that temperature may be a good surrogate for light in establishing control over the dynamics of spiral waves, as was studied previously with optogenetics (22).

## Materials and methods

In order to study the efficacy of temperature-based “attract-anchor-drag” control (22), I used a mathematical model for human atrial tissue. I modeled the time evolution of the transmembrane potential *V* according to Eq. 1.

(1) $$\frac{dV}{dt}=-\frac{I_{ion}+I_{stim}}{C_{m}}$$

Here, $C_{m}$ $\left(\mu F/{\text{cm}}^{2}\right)$ is the specific capacitance of the cell membrane. The net ionic current $\left(I_{ion}\right)$ flowing across the cell membrane was described according to the Courtemanche-Ramirez-Nattel (CRN) model for human atrial cardiomyocytes (32). Thus, $I_{ion}$ is expressed as the sum of 12 ionic currents (see Eq. 3): the fast $Na^{+}$ current $\left(I_{Na}\right)$, the inward rectifier $K^{+}$ current $\left(I_{K1}\right)$, the transient outward $K^{+}$ current $\left(I_{to}\right)$, the ultra-rapid $K^{+}$ current $\left(I_{Kur}\right)$, the rapid and slow delayed rectifier $K^{+}$ currents ($I_{Kr}$ and $I_{Ks}$, respectively), the $Na^{+}$ and $Ca^{2+}$ background currents ($I_{BNa}$, and $I_{CaL}$), the $Na^{+}/K^{+}$ pump current, the $Ca^{2+}$ pump current $\left(I_{pCa}\right)$, the $Na^{+}/Ca^{2+}$ exchanger current $\left(I_{NaCa}\right)$, and the $L-type\;Ca^{2+}$ current $\left(I_{CaL}\right)$.

(2) $$I_{ion}=I_{Na}+I_{K1}+I_{to}+I_{Kur}+I_{Kr}+I_{Ks}+I_{BNa}+I_{BCa}+I_{NaK}+I_{CaP}+I_{NaCa}+I_{CaL}$$

I modified the original CRN model according to Majumder et al. (33) to include temperature effects for the atrial working myocardium. In particular, I used the values listed in Table 1 for the different conductances of the ion channels. This enabled us to obtain an action potential with $APD_{90}$ (37°C) = 284 ms in the healthy state. In order to model the action potential during chronic AF (cAF), I used a parameter set from a previous publication (33). Specifically, I down-regulated the maximum conductances of $I_{to}$ and $I_{CaL}$ by 85 and 74%, increased the peak $I_{K1}$ by 250%, increased the activation time constant for $I_{CaL}$ by 62%, and shifted the fast $Na^{+}$ inactivation curve by +1.6 mV and the activation curves for $I_{to}$ and $I_{CaL}$ by +16 and −5.4 mV, respectively.

**Table 1:** List of conductances used to define the model at 37°C

$G_{X}$ (nS/pF)	Value in healthy atrial working myocardium
$G_{K1}$	0.117
$G_{to}$	0.1652
$G_{Kr}$	0.0294
$G_{CaL}$	0.1238

For my studies in two and three dimensions (2D and 3D), I used a continuum description of the model with each node expressing a cell. Adjacent nodes were coupled through diffusive coupling, and the spatiotemporal evolution of the transmembrane voltage was studied by solving a monodomain reaction-diffusion-type equation (see Eq. 3).

(3) $$\frac{dV}{dt}=\nabla \cdot \left(D\nabla V\right)-\frac{I_{ion}+I_{stim}}{C_{m}}$$

The temporal part of Eq. 3 was solved numerically using forward Euler method with a time step δt=0.02ms. Disregarding the anisotropic fiber structure of the heart in my 2D (cell culture) and 3D (tissue) simulations, I replaced the rank-2 symmetric tensor D with a scalar constant *D* = 0.0025 cm^2^/ms to obtain a conduction velocity (37°C) = 69.75 cm/s. This enabled us to reduce the spatial term in Eq. 3 to a Laplacian, which was then solved using a centered finite difference scheme (spatial resolution: 0.03cm).

Finally, I incorporated temperature scaling into the CRN model by either multiplying the rate constants α(37°C) and β(37°C) of the gating variables by Q(T) or dividing the time constant τ(37°C) by Q(T) (according to the availability of parameter values in the original CRN model description).

(4) α(T)=α(37°C).Q(T)

(5) β(T)=β(37°C).Q(T)

(6) $$Q\left(T\right)={\left[Q_{10}\right]}^{\frac{T-37{}^{\circ}C}{10{}^{\circ}C}}$$

The list of $Q_{10}$ factors that I used in my model are listed in Table 2 (29,33).

**Table 2:** Temperature scaling factors ($Q_{10}$ values) for different ionic currents

For current	$Q_{10}$
$I_{Na}$	3.0
$I_{to}$	2.2
$I_{Kur}$	2.2
$I_{Kr}$	3.3
$I_{Ks}$	2.57
$I_{CaL}$	2.2

To compute the temperature field (T) in 2D and 3D simulations, I solved the time-dependent heat-transfer equation (34):

(7) $$\left(\rho C\right)\frac{\partial T}{\partial t}=\nabla \cdot \left(k_{t}\nabla T\right)\text{.}$$

Here, ρ, *C*, and $k_{t}$ represent, respectively, the density (1050 kg/m^3^), specific heat capacity (4219 J/(kg K)), and thermal conductivity (0.7 W/(m K)) of human cardiac tissue (33). In real cardiac tissue, two very important points to note are 1) internal heating of the tissue through blood perfusion (which would contribute a source term to Eq. 7) and 2) boundary conditions to take into account heat flux into the tissue (due to contact with the warm blood in the cavum of the atrium). In the present “proof-of-concept” study, I consider an isolated slab of tissue, disregarding perfusion. This allows me to ignore the source term in Eq. 7. I maintain the top and bottom faces of the slab at 37°C (except where the spot is applied). Thus, $\hat{n}\cdot \left(k_{t}\nabla T\right)=0$ (Neumann boundaries) on the vertical faces of the domain and T=37°C (Dirichlet boundaries) on the horizontal faces. Spiral and scroll waves were induced using the standard S1-S2 cross-field protocol.

## Results and discussion

**Figure 1:**
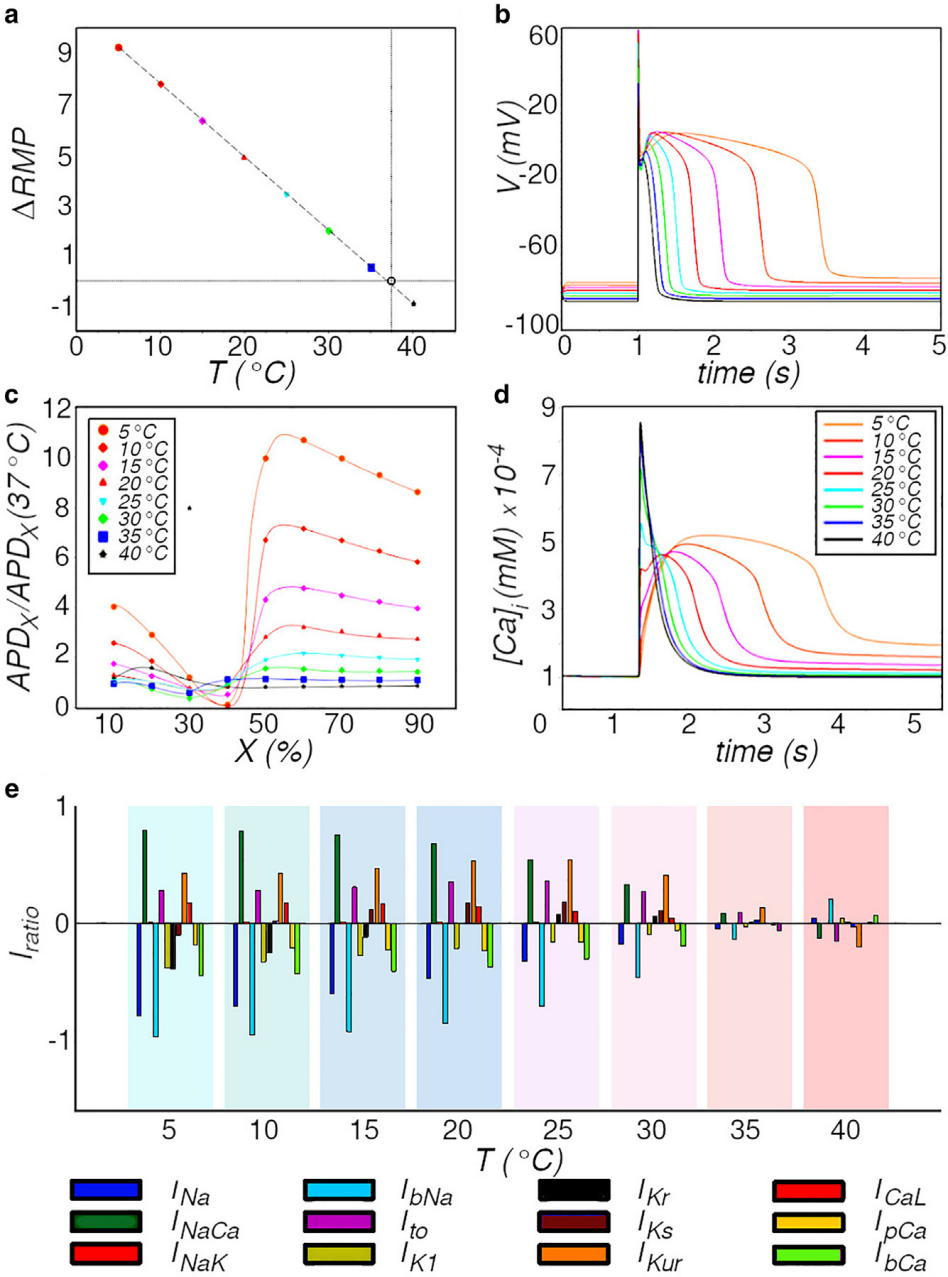
Effect of temperature (T) on the electrophysiological properties of single human atrial cardiomyocytes. (*a*) Action potentials recorded at different *T*. (*b*) *T* dependence of the increase or decrease of action potential duration (APD) at X% repolarization of the cell membrane, relative to the APD at 37°C. (*c*) Change in resting membrane potential $\left(\Delta RMP=RMP_{T{}^{\circ}C}-RMP_{37{}^{\circ}C}\right)$ as a function of *T*. (*d*) Effect of *T* on the ${\left[Ca\right]}_{i}$ transient. (*e*) Relative change $I_{ratio}$ in the peak ionic current (see *bottom* for legend) as a function of *T*. $I_{ratio}=\frac{I_{peak}\left(T\right)-I_{peak}\left(37{}^{\circ}C\right)}{I_{peak}\left(37{}^{\circ}C\right)}$.

In order to determine the strength of the temperature influence on various electrophysiological (EP) properties of cardiomyocytes, I first examined individual heart cells from the healthy atrial working myocardium. Allowing the simulated cell to attain thermal equilibrium at the new temperature, I measured the RMP and APD $\left(APD_{X}\right)$ at different levels of repolarization and the $Ca^{2+}$ transients and peak values of each current participating in the action potential (Eq. 3). I then estimated the impact of temperature on these EP properties by calculating 1) the shift in RMP (ΔRMP) relative to body temperature (37°C) as I decrease *T*, 2) the ratio of $APD_{X}$ at T°C to that at body temperature, and 3) $I_{ratio}$, which is the ratio of change in peak current (increase/positive, decrease/negative) to the value of the peak current at body temperature. These results are presented in Fig. 1.

Fig. 1*a* shows the dependence of ΔRMP on *T*. I find that a shift in *T* from 37°C to 5°C leads to a +9mV shift in RMP. Despite the high degree of nonlinearity in the system, ΔRMP was found to increase linearly with decrease in *T*. Fig. 1 *b* shows single-cell AP obtained from simulated healthy human atrial cardiomyocytes. I observe that a lowering of temperature leads to a drastic change in AP morphology with an associated ninefold increase in the APD at 90% repolarization, sixfold increase at 50% repolarization, and ninefold decrease in APD at 40% repolarization at 5°C. Similar trends are followed at temperatures between 5°C and 40°C. These results are presented in Fig. 1 *c*. Based on these observations, I conclude that temperature has the greatest impact on the AP during phase 3 (plateau). Since the main event occurring during this phase is $Ca^{2+}$ translocation within the cell and across the cell membrane, I expected to see a substantial role of the $Ca^{2+}$ transients and $Ca^{2+}$ currents in shaping the AP. My studies confirmed that $Ca^{2+}$ transients do play a major role (Fig. 1 *d*): the triangular spike characteristic of the ${\left[Ca\right]}_{i}$ at 37°C is converted into a broad rounded bump at 5°C. The maximum concentration of ${\left[Ca\right]}_{i}$ is also nearly halved. The $Ca^{2+}$ currents, on the other hand, change with temperature as well, but the changes were weaker than expected. I observe that the L-type Ca2+ current increases slightly with a decrease in *T*, whereas $I_{pCa}$ and $I_{bCa}$ decrease, as illustrated in Fig. 1 *e*. Quite surprisingly, the impact on other currents was stronger. $I_{Na}$ was found to decrease consistently with decrease in *T*. Peak $I_{Na}$ reduced almost fivefold as the temperature was lowered to 5°C. $I_{Kr}$ first increased as *T* was decreased down to 20°C. Then further decrease in *T* led to a strong decrease in $I_{Kr}$, which could explain the abrupt change in AP morphology from a triangular form to a spike-dome form at temperatures below 20°C. $I_{Ks}$ followed a trend similar to $I_{Kr}$, exhibiting an initial increase with decrease in *T* down to 20°C. A further decrease in *T* results in a reduced increase, followed by an overall decrease in peak $I_{Ks}$, below its value at 37°C. These data are presented in Fig. 1 *e*.

**Figure 2:**
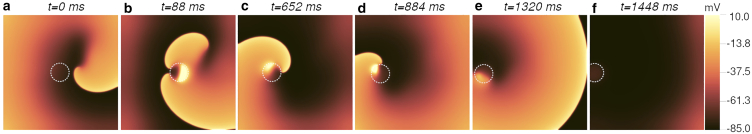
Thermal dragging of a spiral wave. Cells within the cold spot (area encircled by *dashed white line* in subfigures *a–f*) are at 5°C, whereas the rest of the domain is at 37°C. As the spot is moved toward an inexcitable boundary on the left, the spiral follows (*a–d*). Eventually, the phase singularity at its tip collides with the boundary (*e*) and is eliminated (*f*).

Based on my findings at the single-cell level, I conclude that the EP properties of “cold” cardiomyocytes are so different from those of “warm” cardiomyocytes that in extended media, localized cold zones can act as regions of temporary ionic inhomogeneities similar to those induced by light in optogenetics. This means that, if applied strategically, cold spots have the potential to anchor cardiac spiral waves in a manner similar to light spots on optogenetically modified cardiac tissue. In order to test this hypothesis, I induced a spiral wave in a mathematical model of human cardiac tissue with cAF. I then cooled the cells within a circular “spot” of radius 9 mm, close to the tip of the spiral wave. I allowed the system to evolve in space and time. I observed that the spiral wave anchored successfully to the cold spot, as expected (see Fig. 2, *a* and *b*). Next, I shifted the cold spot to the left by 1 cm, as illustrated in Fig. 2 *c*. I allowed the system to reheat the cells within the first spot location back to body temperature while cooling the cells in the new location down to 5°C. I observed that the spiral sensed the shift of the cold zone and moved away from the zone of reheating to anchor to the new location of the cold spot. Thus, it was possible to drag a spiral wave in 2D using a low-temperature spot. Fig. 2, *a*–*e*, illustrates the step-by-step approach to relocate a spiral wave using a cold spot. In this case, I used the spot to drag the spiral wave toward an inexcitable boundary of the domain in favor of termination (Fig. 2 *f*).

Next, I applied the same protocol on a simulated 3D slab of human atrial tissue. First, I simulated a tissue slab with no EP model but with parameters suited to represent thermal properties of cardiac tissue. I observed that a cold spot (5°C) requires about 15−16 s to establish a full transmural profile in a tissue of thickness 0.25 cm.

**Figure 3:**
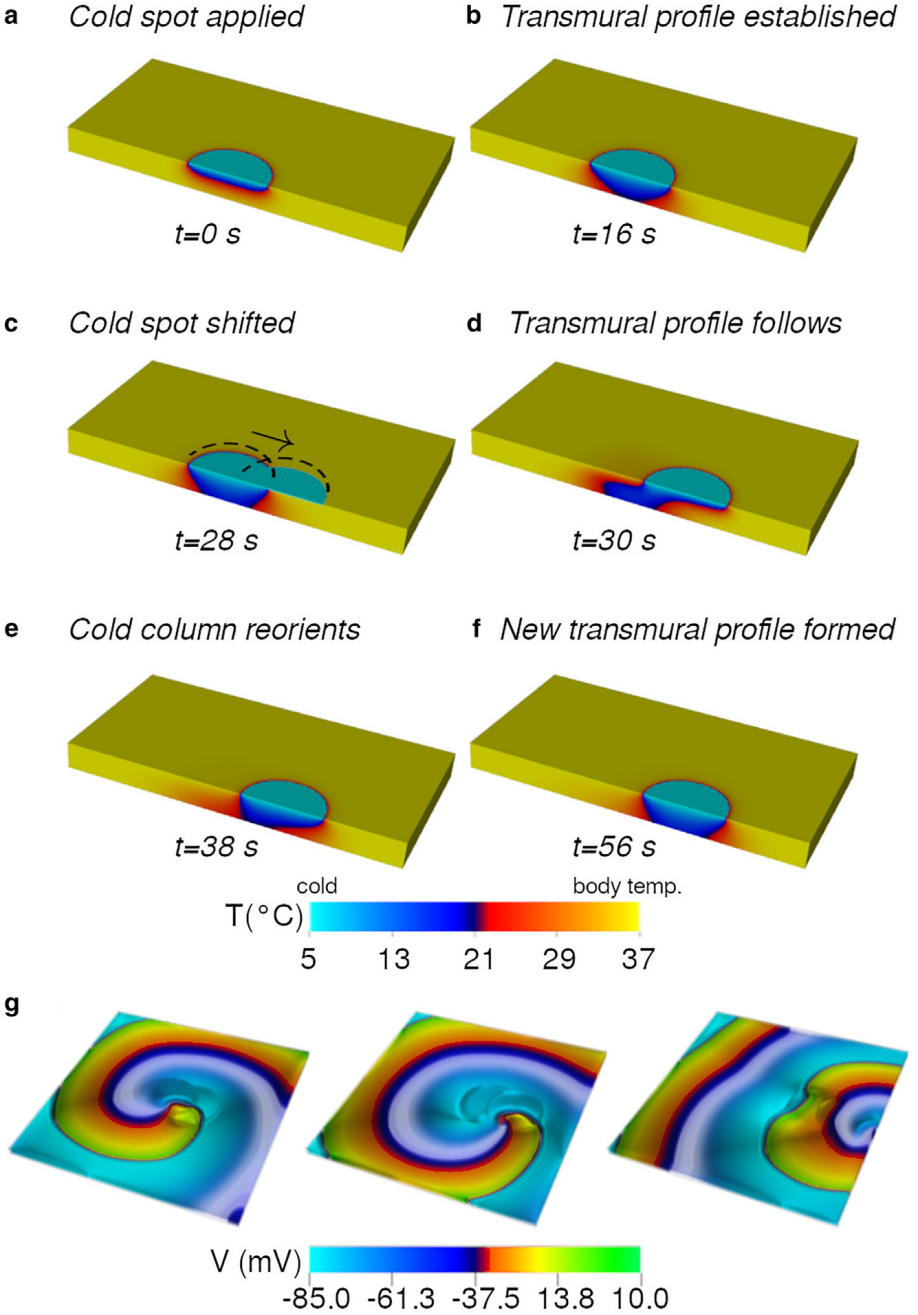
Thermal dragging of a scroll wave. (*a*)–(*f*) show the step-by-step development of the temperature profile inside a 0.25-cm-thick slab of cardiac tissue. A cold spot of temperature 5°C is applied on the upper surface of the slab. The rest of the slab is at body temperature. The temperature profile establishes as a cold column, reaching the full depth of the tissue in 16 s. The cold spot is then moved (indicated by *black arrow*). The cold column reorients itself to establish a new profile within the slab. (*g*) demonstrates the possibility to drag a scroll wave within a 3D slab of cardiac tissue (15 ×15×0.3 cm). Total time from first application of the cold spot to elimination of the scroll is 36 s.

Fig. 3, *a*–*f*, illustrates how the formation of a cold column inside the thickness of the 0.25-cm-thick 3D tissue when a circular spot of radius 9 mm and temperature 5°C is applied to the top face of the slab. Because of the moderate thermal conductivity of cardiac tissue, the cold column (zone with temperature lower than body temperature) requires about 15 s to establish fully transmurally. Once the column is established, cells in each layer of the slab have the potential to anchor the section of the spiral wave in the respective layer. Thus, I expect the filament of the scroll to wrap around the inhomogeneity (the cold column) with ease. When the cold spot is moved, the temperature profile inside the tissue needs to readjust. My studies reveal that a transient distortion is visible in the profile of the cold column (Fig. 3, *c* and *d*). However, given time (see Fig. 3, *e* and *f*), the column straightens itself at the new spot location.

In order to test the feasibility of relocating a scroll wave with the shifting cold column, I coupled Eq. 7 to my EP model in 3D. My studies presented in Fig. 3 *g* prove the principle that dragging a scroll wave is possible with a low-temperature spot applied to the surface of cardiac tissue, provided I allow the system to establish a fully transmural cold column, regardless of its shape. On a tissue of thickness 0.25 cm, it took 36 s to relocate a scroll wave through a distance of 7 cm. Obviously, the thicker the tissue, the longer the timescales involved. These results provide useful insights into the usability of temperature as a possible replacement for optogenetic techniques.

One of the main difficulties with using temperature to control cardiac electrophysiology is that long timescales are involved. Whereas with optogenetics, one can expect to control spiral or scroll wave dynamics within milliseconds, temperature-based control is 1000 times slower and determined by the thermal conductivity of cardiac tissue. Full transmural establishment of the cold column can be achieved within 10−15 s in case of the atria (depending on the wall thickness). However, relocation toward an inexcitable boundary can take up to minutes depending on how far the scroll needs to be dragged for control. For ventricles, which are generally thicker than atria, the timescales are even longer. In 2D, this does not pose a problem because a transmural gradient of temperature is not required. As shown in Fig. 2, anchoring occurs within 1–4 rotations of the spiral. Similar efficacy can be expected in thin regions of the atrial wall. In 3D, however, scroll dragging is possible if, and only if, the scroll filament is pinned to the thermally induced inhomogeneity inside the tissue, as it is on the surface. Thus, establishment of the transmural cold column is necessary. For the establishment of a full transmural profile in a tissue slab of thickness 0.25 cm, we have to wait for >10 s. If we include internal heating, the required time would be even longer (of the order of minutes).

In a previous study, we discussed the effect of global cooling of the heart in the presence of ongoing spiral wave activity. Such cooling can have both stabilizing and destabilizing effects on the wave dynamics. However, here, I deal with localized cooling, which affects only a small region of the tissue at any given time. Thermal gradients are produced inside the tissue as shown in Fig. 3. Any subsurface layer contains a central circular spot of low temperature, surrounded by concentric rings of gradually increasing radius and increasing temperature. This thermal pattern propagates transmurally to eventually establish the cold column. According to Fig. 1, cooling causes monotonic slowdown of all subcellular processes, resulting in the prolongation of APD, elevation of RMP, etc. Therefore, in Fig. 3, the temperature distribution in each subsurface layer gives rise to an ionic inhomogeneity with longer APD inside compared to outside. If the inhomogeneity is created close to the core of the spiral in the particular layer, then the tip is trapped and forced to anchor. Thus, thermal gradients produce a stabilizing effect in this case. My results show that a fully transmural—but not necessarily straight—cold column is required to drag a scroll wave. If the scroll filament is properly anchored to the cold column, then moving the spot on the surface of the slab can cause relocation. Anchored scroll filaments elongate when the cold column distorts during relocation. Brief detachments could also be observed. However, such phases are transient and disappear as soon as the temperature profile stabilizes. Consequently, if the cold spot is brought close to an inexcitable boundary of the slab, then the scroll filament can be removed as well.

Therapeutic hypothermia is already practiced in the clinic on patients who have suffered from cardiac arrest. It involves slowly cooling the heart to 32°C–36°C over a period of 12–24 h (35). The cooling can be external or endovascular. The latter method is considered safe and quick (36). In the present study, I require local cooling of the tissue. Therefore, endovascular cooling is more appropriate. However, unlike therapeutic hypothermia, the proposed approach requires much lower temperatures (0°C–5°C), making it, in principle, closer to cryoablation. At this temperature, the cellular electrophysiology is profoundly affected, but the membrane proteins are not. As long as the cells are not frozen, normal function can be restored upon rewarming (37). Previous studies dedicated to investigating the mechanisms of fibrillation during hypothermia show that low temperatures can induce wave breaks and sustained reentry if the heart is cooled as a whole (38). My approach relies on cooling small regions of the heart that are believed to lie close to the source of the arrhythmia. Thus, the probability of inducing wave breaks is very low. Thus, cooling provides us with the ideal temporary, reversible inhomogeneities that are necessary for anchoring of spiral waves and opens up new horizons to cardiac arrhythmia research.

## Conclusion

I have upcycled an innovative approach to control cardiac arrhythmias in the heart using low supra-zero temperatures. This approach scores over all previous established techniques in that it does not involve the administration of drugs, genetic modification of the heart (as opposed to opto- and sonogenetics), injection of foreign particles (e.g., biocompatible electrodes), application of high voltage shocks (as in conventional defibrillation), or inflict damage to excitable cardiac tissue (as is done with ablation).

To demonstrate the feasibility of the approach, I first investigated the underlying EP mechanisms at the single-cell level. Then, I tested the technique in 2D on simulated cardiac tissue. The success of the technique in 2D motivated us to explore its applicability in 3D. This proof-of-principle study lays the foundation of a new field of research concerning thermodynamic control of spiral waves in cardiac tissue systems. My studies revealed that such control is executed provided the cold column is fully transmural. Finally, while the approach appears to be the most sustainable one, given its simplicity and minimalistic nature, it is yet to be determined if its efficacy would be comparable to existing clinical practices such as antitachycardia pacing, a clinically relevant approach for efficient spiral wave control by local overdrive pacing. Such a study would involve a statistically large number of experiments on tissue slabs with different pacing frequency versus speed of thermal relocation, different pacing amplitudes versus temperature of applied spot, etc. Thus, such a comparative study lies beyond the scope of the present manuscript and is being considered for a future publication.

## Author contributions

R.M. designed the research, carried out all simulations, analyzed the data, prepared the figures, and wrote the manuscript.
